# Impacts of poultry by-product meal substituting fishmeal on growth efficiency, body composition, liver, and intestine morphology of European sea bass, *Dicentrarchus labrax*

**DOI:** 10.1016/j.fochx.2024.101569

**Published:** 2024-06-16

**Authors:** Yasser Marzouk, Magdy M. Gaber, Ishtiyaq Ahmad, Imtiaz Ahmed, Mohammed F. El Basuini, Mohamed Abdullah Zaki, Abd-Elaziz M. Nour, Eman M.H. Labib, Hala Saber Khalil

**Affiliations:** a Animal Production Department, Faculty of Agriculture, Alexandria University, Alexandria, Egypt; b National Institute of Oceanography and Fisheries, Alexandria, Egypt; c Fish Nutrition Research Laboratory, Department of Zoology, University of Kashmir, Srinagar 190006, India; d Division of Fish Genetics and Biotechnology, Faculty of Fisheries, Rangil, Ganderbal, SKUAST-Kashmir, 190006, India; e Animal Production Department, Faculty of Agriculture, Tanta University, 31527, Tanta, Egypt; f Faculty of Desert Agriculture, King Salman International University, South Sinai 46618, Egypt; g Utilization of By-Products Department, Animal Production Research Institute, Agriculture Research Center, Ministry of Agriculture and Land Reclamation, Dokki, Giza, Egypt; h Aquaculture Department, Faculty of Fish Resources, Suez University, Suez, 43221, Egypt; i College of Fisheries and Aquaculture Technology, Arab Academy for Science, Technology, and Maritime Transport, Alexandria, Egypt

**Keywords:** Sea bass, Poultry by-products, Amino acids, Growth efficiency

## Abstract

A twelve week feeding experiment was conducted to evaluate the replacement of fishmeal (FM) with poultry by-product meal (PBM) in practical diets for European sea bass, *Dicentrarchus labrax* with an average initial weight of 0.89 g. Five isocaloric (5.1 kcal lipid g^−1^) and isonitrogenous (451 g protein kg^−1^) diets were formulated with PBM replacing FM at levels of 0% (control), 25%, 50%, 75%, and 100%. The experiment was carried out in 30-in. nylon mesh net cages (hapas). At the termination of the trial, growth performance including final body weight, weight gain, specific growth rate, and protein growth rate of diets containing up to 75% PBM were comparable to those of the control group, whereas the diet with 100% PBM resulted in a significantly lower values (*p* < 0.05). Feed utilization exhibited variation among the treatments (*p* < 0.05). Whole body composition also showed significant differences across the dietary treatments. Essential amino acid (EAA) contents specifically arginine (Arg), histidine (His), methionine (Met), and threonine (Thr) in the whole body of fish fed diets with up to 50% PBM replacement were not significantly different from those in the control group. Furthermore, the intestinal microvilli length, width and absorption area increased significantly (*p* < 0.05) with PBM replacement levels up to 50%. Histological analysis of the liver revealed mild vacuolation of hepatocytes in fish fed up to 50% PBM,while pre-pancreatic fatty degeneration of hepatocytes was observed in fish fed diets with 75% and 100% PBM. Therefore, this study demonstrates that PBM can replace up to 50% of FM in the diets of European sea bass without adverse effects on growth performance, body composition, or liver and intestine morphology.

## Highlights

• Up to 75% PBM replacement for FM maintains growth, but 100% replacement significantly reduces it.
• Intestinal morphology improved with Up to 50% PBM replacement improved intestinal morphology, however higher levels caused mild liver vacuolation and fatty degeneration.
• Overall, the study suggests that PBM can replace up to 50% of FM in the diets of European sea bass without adverse effects on growth rate.

## 1 Introduction

Diet costs represent the largest annual varyiable expenditure in aquaculture, accounting for 70–80% of operational costs throughout production (Allam et al., 2020). In the context of European sea bass, *Dicentrarchus labrax* profitability, feed costs are particularly critical, constituting 60–70% of total production expenses (Hussein et al., 2023; Rawles et al., 2011). Consequently, evaluating cost-effective and nutritionally efficient diets is essential for enhancing the profitability of the sea bass sector. There are two main strategies to accomplish this, mainly by evaluating lowering crude protein levels and substituting expensive marine fishmeal with different sources of protein (Rawles et al., 2011).

Animal protein sources such as fishmeal (FM) and poultry by-product meal (PBM) are the primary sources of protein in animal feeds (Chaklader, Siddik, & Fotedar, 2020). Fishmeal despite being a highly acceptable protein source for European sea bass produced in re-circulating aquaculture systems (RAS); remains the most expensive ingredient in macro feed and is highly sought after by other livestock industries (Siddik, Howieson, & Fotedar, 2019; Thompson et al., 2008). Any unfavorable market disruption, supply disruption, or availability issue might result in sharp rise prompt in the commodity price for clupeid fish populations that are harvested for fishmeal (Rawles et al., 2011; Thompson et al., 2008). Additionally, many experts agree that the current rate of wild fish harvesting for cultured fish feed is unsustainable at the current rate (Naylor et al., 2000). In contrast, poultry by-product sources may offer a more cost-efficient alternative to fishmeal (Dawson, Alam, Watanabe, Carroll, & Seaton, 2018; Siddik et al., 2019).

Poultry by-product meal (PBM) derived from chicken hatcheries has a great amount of protein about 55–60%, offers a balanced amino acid profile, and is less costly than fishmeal (Dawson et al., 2018). Like soybean meal, bone meal and meat meal, dietary PBM is effectively digested by various aquatic animals. For instance, *Psetta maeotica*, a black sea turbot exhibited reduced growth when fed diets containing >25% PBM instead of fishmeal (Yigit et al., 2006). Conversly *Nibea miichthioides*, a cuneate drum was fed successfully on a diet that replaced half of the fishmeal with PBM (Wang, Guo, Bureau, & Cui, 2006). Kureshy, Davis, and Arnold (2000) successfully reared juvenile red drum (*Sciaenops ocellatus*) on diets substituting 66.7% of fishmeal with PBM, suggesting that even higher levels of substitution might be feasible with further research.

The successful use of PBM in the diets of freshwater fish species has also been documented. For instance, Nile tilapia (*Oreochromis niloticus*) exhibited no negative growth impacts when fishmeal was completely replaced with PBM (Sing, Kamarudin, Wilson, & Sofian-Azirun, 2014). Similarly, Muzinic, Thompson, Metts, Dasgupta, and Webster (2006) successfully replaced all dietary fishmeal with turkey meal without adversely affecting the growth performance of sunshine bass (*Morone chrysops* x *Morone saxatilis*). Moreover, diets incorporating 50% PBM in place of fishmeal supported good growth in Gibel carp (*Carassius auratus gibelio*) (Yang et al., 2004).

European sea bass is a significant species in the aquaculture industry, particularly popular in Mediterranean and Southern European countries (Carter, Mente, Barnes, & Nengas, 2012; Llorente et al., 2020). This species has garnered considerable attention due to advancements in aquafeeds that can be used from the start of feeding without compromising growth performance compared to live feeds (Guillaume, Kaushik, Bergot, & Metailler, 2001; Joshua, Kamarudin, Ikhsan, Yusoff, & Zulperi, 2022). However, there is a notable gap in research on the use of PBM as a replacement for fishmeal during the grow-out phase of European sea bass.

The primary objective of our research is to evaluate the substitution of fishmeal (FM) with poultry by-product meal (PBM) in the diets of European sea bass and its effects on production efficiency, body composition, and liver and intestine morphology. This study aims to provide insights that could enhance the sustainability and profitability of European sea bass aquaculture.

## 2 Materials and methods

This experiment was designed to assess the effects of replacing fishmeal (FM) with poultry by-product mea (PBM) on growth efficiency, body composition, and liver, and intestine morphology of European sea bass, *Dicentrarchus labrax* over a period of 12 weeks.

### 2.1 Ethical statement

The study adhered to the applicable global standards for handling experimental animals as approved by the Faculty of Desert Agriculture, King Salman International University, Egypt (KSIU/2023/DA-1). All methods in this study were performed in accordance with the relevant guidelines of ARRIVE.

### 2.2 Experimental diets

In this study, five isonitrogenous (451 g protein kg^−1^) and isocaloric (5.1 kcal lipid g^−1^) diets were formulated to contain varying levels of poultry by-product meal (PBM) as a replacement for fishmeal: D0% (Control diet), D25% (25% PBM), D50% (50% PBM), D75% (75% PBM), and D100% (100% PBM)(Table 1). These diets were designed to meet the nutritional requirements of European sea bass as specified by NRC (2011). The PBM was produced at 80 °C for 10 h from dead chickens at the hatchery supplemented with a mixture of 10 amino acids. All dry components were thoroughly mixed. PBM, vitamin mix, mineral mixtures and fish oil were separately mixed and gradually added to the dry mixture. The remaining components were finely ground into a powder using a blender to prepare the experimental diets. The ingredients were mixed together, and warm distilled water (40 °C) was slowly added. The mixture was then extruded into pellets with a diameter of 2.5 mm, air dried, and stored in sealed polythylene bags until feeding. Before feeding, the diets were thawed and broken into small pieces as required. Diets with 25–100% PBM were supplemented with an amino acid mixture to increase the crude protein content to 45%. The proximate compositions of the feed ingredients are presented in Table 1, while the amino acid profile of the experimental diets was analyzed using Biochrom 30+ Amino acid analyzer with the results shown in Table 2.

**Table 1:** Feed formulation and proximate composition of diets comprised PBM fed to European sea bass (*D. labrax L*) for 12 weeks.

Ingredients (%)	Level of replacement (%)				
	D0%	D25%	D50%	D75%	D100%
Fishmeal (C P 65%)	50.0	37.5	25.0	12.5	–
Poultry by-product meal (PBM)^a^	–	12.5	25.0	37.5	50.0
Soybean meal (C P 44%)	19.0	19.0	19.0	19.0	19.0
Yellow corn meal	7.4	7.4	7.4	7.4	7.4
Rice bran	11.7	11.7	11.7	11.7	11.7
Fish oil	3.0	3.0	3.0	3.0	3.0
Sunflower oil	3.0	3.0	3.0	3.0	3.0
Vit. & Min. premix^b^	2.0	2.0	2.0	2.0	2.0
Calcium diphosphate	1.0	1.0	1.0	1.0	1.0
Molasses	2.0	2.0	2.0	2.0	2.0
Cholin chloride	0.2	0.2	0.2	0.2	0.2
Vitamin C	0.3	0.3	0.3	0.3	0.3
Antitoxins	0.3	0.3	0.3	0.3	0.3
Enzymes (Pepsin)	0.1	0.1	0.1	0.1	0.1
**Proximate analyses (%)**					
Moisture	6.2	6.1	6.1	5.8	5.76
Crude protein	45.1	45.2	45.1	45.0	45.1
Crude lipid	18.3	18.4	18.3	18.2	18.4
Ash	7.7	7.9	7.5	7.8	7.5
Crude fibre	3.2	3.8	3.8	3.4	3.5
NFE^c^	19.5	18.6	19.2	19.7	19.7
Gross energy (kcal g^−1^)^d^	5.1	5.1	5.1	5.1	5.1
P:E ratio (mg CP^−1^ kcal^−1^)^e^	93.0	94.4	94.4	92.8	93.3

a Poultry by-product meal (PBM) composed from chicken dead +10 amino acids mixture.

b Vitamin and mineral premixed: Vitamin premix (kg of premix): Vit. B2 (1450.0 mg), Vit. B1 (1650.0 mg), Vit. B6 (2000.0 mg), Biotin (20.0 mg), Inositol (1000.0 mg), Calcium pantothenate (1000.0 mg), Niacin (1000.0 mg), Vit. C (1000.0 mg), Vit. D3 (150,000 IU), Folic acid (600.0 mg), Choline chloride (300.000 mg), Vit. A (500,000 IU), Vit. B12 (600.0 mg), α-tocopherol acetate (3000.0 mg), and Vit. K3 (450.0 mg); Mineral premix (g kg^−1^ of premix): Manganese (6500.0 mg), Zinc (5000.0 mg), Iron (3000.0 mg), Copper (1700.0 mg), Iodine (450.0 mg), Selenium (65.0 mg), Cobalt (60.0 mg) and Calcium carbonate (as carrier) (up to 1 kg).

c NFE (%): Nitrogen Free Extract =100 - (CP % + CL% + CF % + ash %).

d The conversion factors 5.65, 9.45, and 4.1 kcal g^−1^ were used to calculate the calorific values for protein, lipid, and carbohydrate, respectively.

e P/E ratio = Protein to energy ratio mg crude protein/Kcal gross energy.

**Table 2:** Analyzed amino acid contents of investigational diets contained PBM fed to European sea bass (*D. labrax L*).

	Level of replacement (%)					
Amino acids	D0%	D25%	D50%	D75%	D100%	Required^a^
Arginine Histidine Isoleucine Leucine Lysine Methionine Phenylalanine Therionine Valine Tryptophan	2.80 1.01 2.11 3.22 3.21 1.10 1.90 1.70 2.30 0.50	2.82 1.02 2.11 3.01 3.00 1.10 1.81 1.71 2.21 0.51	2.83 1.03 2.03 3.03 2.70 1.18 1.88 1.63 2.23 0.72	2.81 1.02 1.92 3.01 2.41 1.11 1.72 1.53 2.24 0.71	2.82 0.91 1.81 2.81 2.11 1.12 1.63 1.41 2.11 0.83	1.39 1.61 0.47 2.41 2.27 1.09 1.43 0.61 1.22 0.42

a According to Gaber et al. (2015), (*n* = 3).

### 2.3 Fish sampling and investigational setup

A total of 300 European sea bass larvae were obtained from the Marine General Authority for Fish Resources Development (GAFRD) Hatchery, in Alexandria, Egypt. The fish were acclimated at the Fish Nutrition Laboratory, El-Max station, National Institute of Oceanography and Fisheries, Alexandria, Egypt, in three concrete ponds (8.00 × 2.00 × 1.00 m) for 15 days. Initially, the fish were treated with a potassium permanganate (KMnO_4_) dip to eliminate any infections. During acclimatization, the fish were transitioned from live food to an artificial diet. The experiment was conducted in 15 hapas with dimension (1.0 m × 1.0 m × 1.0 m) placed in three cement tanks. The control diet was fed to the experimental fish for two weeks as an adaptation phase. Following this period, each hapa was randomly stocked with 20 European sea bass with triplicates for each treatment.

Fish in each hapa were fed at a rate of 15% of body weight twice daily in the morning and evening hours (6 d a week) for the first 30 days, followed by 12% for 15 days, then 10% and 8% for the remaining days of the trial. The experiment lasted for 12 weeks. During the experimental period, fish were fasted for one-day each week to measure growth performance, and the hapas were cleaned to prevent the accumulation of feces and algae. The tanks were refilled with the same amount of fresh seawater. The study was conducted with a 12-h light / 12-h dark cycle.

### 2.4 Water quality analysis

Physicochemical properties of water were monitored during the entire feeding trial. A YSI Model 58 oxygen meter, Yellow Springs OH USA was used to test water temperature and dissolved oxygen on alternate days. Moreover, ammonia and nitrite were measured by ammonia test kit API at weekly time. Alkalinity was measured twice weekly using Golterman, Clymo, and Ohnstad (1978) titration procedure and electronic pH meter (Oakton pH 620) was used to check pH. During the feeding trial, water temperature was recorded as 27.20 ± 1.80 ^o^ C, dissolved oxygen was 6.80 ± 1.40 mg L^−1^, pH was 7.40 ± 0.61, ammonia was 0.04 ± 0.02 mg L^−1^, nitrite was 0.10 ± 0.05 mg L^−1^, nitrate was 1.50 ± 0.20 mg L^−1^, alkalinity was 1814 ± 6.00 mg L^−1^ and salinity was 35.20 ± 1.10 g kg^−1^. Comparable findings were observed by Sedgwick (1995).

### 2.5 Growth efficiency and feed utilization

All fish weighed and counted as they were transferred from the hapas, to get data about growth parameters according to these formulae:

$$\text{Weight gain}\;\left(g\;{\text{fish}}^{-1}\right)=\mathrm{FBW}-\mathrm{IBW},\mathrm{as}\;\mathrm{IBW}=\text{initial body weight};\mathrm{FBW}=\text{Final body weight}.$$

$$\mathrm{ADG}={\mathrm{FBW}}_{84}–{\mathrm{IBW}}_{0}/\text{experimental period}.$$

$$\text{Specific growth rate}\;\left(\mathrm{SGR}\right)\;\left(\%d^{-1}\right)=100\;\left(\ln\;{\mathrm{FBW}}_{84}–\ln\;{\mathrm{IBW}}_{0}\right)/\text{experimental period}.$$

$$\text{Feed conversion rate}=\mathrm{FI}\;\left(\text{Feed intake};g\right)/\mathrm{WG}\;\left(\text{Weight gain};g\right).$$

$$\text{Energy utilization}=100\;x\;\left(\text{E2}-\text{E1}\right)/\mathrm{EI}.$$

Where E1 and E2 are the energy content of primary and final fish, separately and EI is the energy intake.

$$\text{Protein growth ratio}\;\left(\mathrm{PGR}\right)\;\left(\%\right)=100\mathrm{lnP}2\mathrm{lnP}1/T.$$

Where P1 and P2 are the primary and end protein content in fish, separately, and T is the experimental period.

$$\text{Protein productive value}=\mathrm{PG}\;\left(\text{Protein gain}\right)/\mathrm{PI}\;\left(\text{Protein intake};g\right).$$

$$\text{Protein efficacy rate}=\mathrm{WG}\;\left(g\right)/\mathrm{PI}\;\left(\text{Protein intake};g\right).$$

### 2.6 Chemical analysis of investigational diets and carcass composition of fish

Standard methods were employed to determine the proximate composition of the prepared diets and fish carcasses according to AOAC (2012). Five fish from each hapa were randomly selected at the start and end of the feeding trial and anaesthetized with phenoxyethanol at a concentration of 5 mg L^−1^ for euthanasia to analyze body chemical composition. The specimens were then dehydrated for 24 h in an oven at 105 °C to determine the dry matter (DM), and ash content was measured after incineration at 550 °C for 12 h. Crude protein content was determined using micro-Kjeldahl method, withnitrogen content (N%) converted to protein content by multiplying by 6.25 (using a Kjeltech auto analyzer). Crude lipid content was measured via Soxhlet extraction with diethyl ether (40–60 °C). Crude fibre (CF) content was quantified after a 15 min of digestion process with 5% H_2_SO_4_ and 5% NaOH, followed by drying and ashing. The nitrogen free extract NFE was calculated using the formula: NFE (%) = 100 – (crude protein; CP% + crude lipid; CL% + crude fibre; CF% + ash %). Moreover, at the termination of the study, five fish were selected from each hapa for amino acid profile analysis, and the results are summarized in Table 6.

### 2.7 Histological analysis of liver and intestine

For histological analysis of liver and intestine (midgut) samples, five fish were randomly selected from each treatment (*n* = 5 × 5 = 25) and dissected. The samples were fixed in Bouin's solution (Heyderman, 1992) for 24 hoursfollowed by dehydration through a graded ethanol series, clearing in xylene, and embedding in paraffin wax. Sections were cut at 5–7 μm thinkness and stained with haematoxylin and eosin (H + E) for microscopic examination. The stained slides were then dehydrated through a descending ethanol series cleared in xylene and mounted in Dibutylphthalate Polystyrene Xylene (DPX). The stained sections of the liver and midgut were examined under a digital microscope (Leica DM500) equipped with Image-Pro Plus software and images were captured. Digitized images were analyzed to measure villi length, villi width and villus area, following the procedures described by Escaffre, Kaushik, and Mambrini (2007).

### 2.8 Statistical analysis

A one-way analysis of variance (ANOVA) was conducted to evaluate growth performance metrics, followed by Duncan's multiple range test (Duncan, 1955). The polynomial contrast method (Yossa & Verdegem, 2015) was employed to detect significant (*p* ≤ 0.05) differences among the treatments. Prior to analysis, all percentage and ratio data were transformed using the arcsine square root transformation (Zar, 1984). Polynomial regression analysis was utilized for growth performance parameters. Statistical analyses were performed using Origin software (version 8.5.1; OriginLab Corporation, San Clemente, CA, USA).

## 3 Results

### 3.1 Growth efficiency

**Fig. 1:**
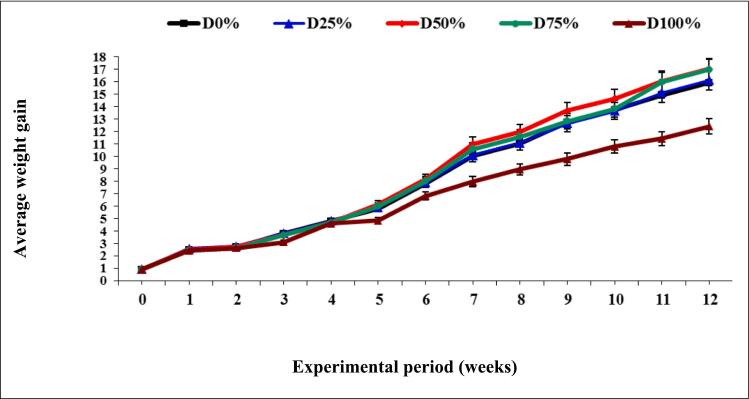
Impact of fishmeal substitution with PBM on growth period of European sea bass (*D. labrax L*) for 12 weeks, *n* = 6.

**Fig. 2:**
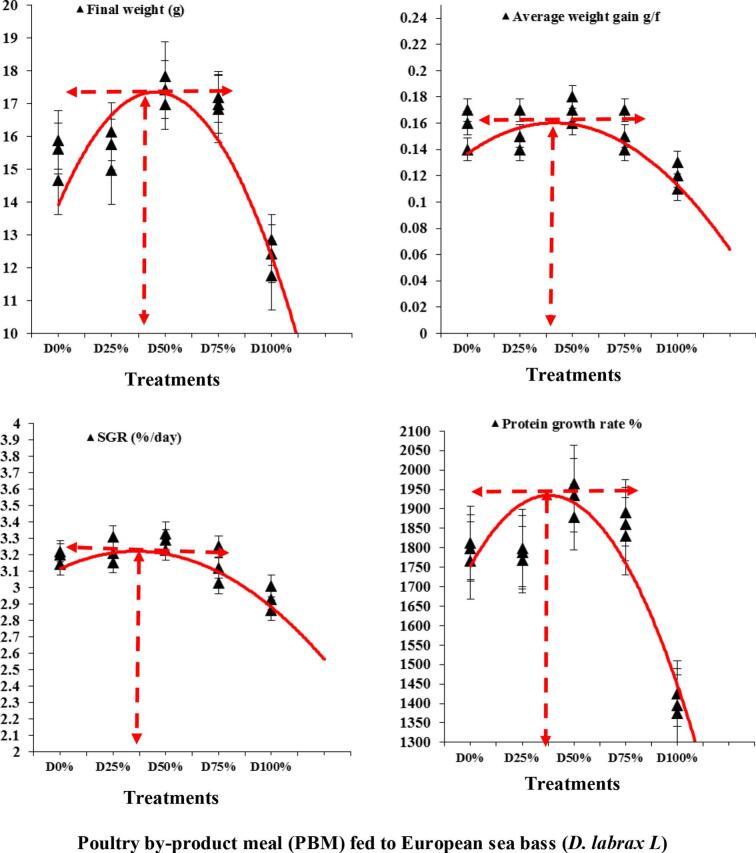
The relationship between final weight (g), average weight gain (g f^−1^), specific growth ratio (SGR; % day^−1^), and protein growth ratio (%) of European sea bass (*D. labrax L*) fed on dietary PBM as fishmeal substitution for 12 weeks.

At the start of the trial (day 0) the initial fish weight ranged from 0.88 ± 0.02 g) with no significant differences (*p* ≥ 0.05) observed across all diets (Table 3,Fig. 1**)**. By day 28, the mean fish weight ranged from 4 to 4.6 g with significant (*p* ≤ 0.05) differences noted. Fish fed the 50% PBM diet were significantly larger than those fed the 100% PBM diet. However, there were no significant differences between the basal diet and the PBM diets. On day 42, the mean fish weight ranged from 5.2 to 5.6 g, with significant (p ≤ 0.05) differences among the diets. By day 56, the fish weight ranged from 6.4 to 8.3 g, with the 100% diet group have significantly (p ≤ 0.05) lower weights compared to the50% PBM. On day 70, the mean fish weight ranged from 9.8 to11.7 g. Fish fed the 100% PBM diet had significantly (*p* ≤ 0.05) lower weights compared to the 50% PBM group and other treatments. By the end of the trial (day 84), the final body weight (FBW) ranged from 12.6 to 16.2 g. The mean final body weight for the 100% PBM group was significantly lower than the other PBM-fed groups, while no significant (*p* ≥ 0.05) differences were noted among the diets containing 0, 25, 50, and 75% PBM (Table 3, Fig. 1). The PBM levels significantly influenced the final weight (g), weight gain (g), specific growth ratio (SGR; % day^−1^), and protein growth ratio (PGR %) of European sea bass (*D. labrax L*) with up to 25–50% fishmeal replacement (*p* ≤ 0.05). The highest values for FW, WG, SGR, and PGR levels were observed in the 50% PBM group (Table 3, Fig. 2**)**. Second-order polynomial regression analysis of growth performance and feed utilization indicated that 50% PBM was the optimal level for replacing fishmeal.

**Table 3:** Impact of fishmeal substitution with PBM on growth efficiency of European sea bass (*D. labrax L*) for 12 weeks.

Level of replacement	Average body weight (g fish^−1^)		Weight gain	ADG	SGR	PGR	Survival
(%)	IBW	FBW	(g fish^−1^)	(g fish^−1^)	(% day ^−1^)	(%)	(%)
D0%	0.88 ± 0.03	15.92 ± 1.40^a^	15.07 ± 1.61^a^	0.17 ± 0.01^a^	3.22 ± 0.10^a^	1813 ± 182.0^a^	95.00 ± 5.00^a^
D25%	0.90 ± 0.02	16.12 ± 0.90^a^	15.27 ± 0.81^a^	0.17 ± 0.01^a^	3.21 ± 0.10^a^	1789 ± 63.0^a^	90.00 ± 5.00^b^
D50%	0.88 ± 0.05	17.04 ± 1.70^a^	16.22 ± 1.80^a^	0.18 ± 0.02^a^	3.29 ± 0.11^a^	1935 ± 175.0^a^	90.00 ± 5.50^b^
D75%	0.88 ± 0.02	16.96 ± 1.31^a^	15.56 ± 1.52^a^	0.17 ± 0.02^a^	3.25 ± 0.12^a^	1860 ± 138.0^a^	95.00 ± 5.00^a^
D100%	0.89 ± 0.03	12.42 ± 0.91^b^	11.53 ± 0.92^b^	0.13 ± 0.01^b^	2.93 ± 0.10^b^	1395 ± 85.0^b^	80.00 ± 5.00^c^

Values are mean followed by standard deviation (±SD). Values in the same column with same superscripts are not significantly different (P˃0.05), (n = 3).

### 3.2 Survival (%)

The effect of poultry by-product meal (PBM) replacement of fishmeal (FM) on survival rates (%) is presented in Table 3. The highest survival rates were observed in fish fed diets with 0% and 75% PBM with no significant differences between these two groups. However, both groups exhibited significant (*p* ≤ 0.5) differences in survival rates compared to the groupsfed 25%, 50% and 100% PBM. The lowest survival rate was recorded in the group fed the 100% PBM diet.

### 3.3 Feed utilization efficiency

The influence of fishmeal (FM) replacement with poultry by-product meal (PBM) on feed consumption across various dietary treatments is presented in Table 4. Fish fed diets containing 0% to 75% PBM (D0 - D75%) exhibited the highest feed intake (FI) (g fish^−1^) compared to those fed the 100% PBM diet, which showed the lowest FI value and these differences were significant (*p* ≤ 0.5) with other treatments. Additionally, the lowest feed conversion ratio (FCR) was observed in the 50% PBM (D50%) group, while the highest FCR was recorded in the 100% PBM (D100%) group. Significant differences (*p* ≤ 0.5) were also noted in protein productive value (PPV), protein efficiency ratio (PER), energy utilization (EU) and energy gain (EG) across the different dietary treatments.

**Table 4:** The impact of replacement fishmeal with PBM on feed utilization of European sea bass (*D. labrax* L) for 12 weeks.

Level of replacement (%)	Feed intake (g fish^−1^)	FCR	PER	PPV (%)	Energy gain (Kcal)	Energy utilization (kcal 100 g^−1^)
D0% D25% D50% D75% D100%	20.40 ± 0.20^a^ 20.70 ± 0.80^a^ 20.70 ± 0.40^a^ 21.00 ± 0.60^a^ 19.50 ± 0.40^b^	1.37 ± 0.14^a^ 1.36 ± 0.10^a^ 1.29 ± 0.16^a^ 1.36 ± 015^a^ 1.70 ± 0.12^b^	1.63 ± 0.20^b^ 1.64 ± 0.10^b^ 1.74 ± 0.20^a^ 1.65 ± 0.20^b^ 1.31 ± 0.10^c^	25.85 ± 4.27^b^ 32.77 ± 1.81^a^ 33.23 ± 3.68^a^ 25.96 ± 2.34^b^ 26.04 ± 2.66^b^	25.02 ± 4.68^b^ 30.05 ± 1.83^a^ 30.07 ± 4.58^a^ 24.94 ± 1.56^b^ 21.54 ± 1.34^c^	24.11 ± 4.32^b^ 28.92 ± 2.77^a^ 28.88 ± 4.52^a^ 23.52 ± 2.05^b^ 21.77 ± 1.18^b^

The mean is followed by the standard deviation (SD). Values in the same column with the same superscripts do not differ significantly (P˃0.05), (n = 3).

### 3.4 Whole body composition

Significant (*p* ≤ 0.05) changes in whole body composition were observed at the end of the feeding trial, as shown in Table 5. The results indicated that whole body dry matter, ash and energy content were significantly (*p* ≤ 0.01) affected by the replacement of fishmeal with PBM. Similarly, crude protein content varied significantly (*p* ≤ 0.05) among the treatments with the highest value observed in the D0% PBM group. However, lipid content did not show significant differences across treatments except in the D0% PBM group, which recorded the highest lipid content. Fish fed the D75% PBM diet had the highest ash content, followed by those fed the 25% and 50% PBM diets. Conversly, fish fed the D75% PBM diet had the lowest carcass energy content, whereas fish fed the D0% diet had the highest energy content.

**Table 5:** Impact of fishmeal substitution with PBM on whole body composition (dry weight basis) of European sea bass (*D. labrax L*) for 12 weeks.

Level of replacement (%)	Dry matter (%)	Protein (%)	Lipid (%)	Ash (%)	Carcass energy (kcal 100 g^−1^)
D0%	27.60 ± 2.50^b^	60.40 ± 3.61^a^	28.10 ± 1.50^a^	14.60 ± 1.21^c^	581.60 ± 9.50^a^
D25%	33.90 ± 0.10^a^	57.50 ± 3.91^b^	25.00 ± 3.70^b^	16.30 ± 0.31^b^	560.30 ± 12.10^b^
D50%	32.40 ± 1.40^a^	57.60 ± 2.71^b^	24.20 ± 2.50^b^	17.00 ± 0.31^b^	553.10 ± 8.90^b^
D75%	28.91 ± 2.11^b^	56.10 ± 1.81^b^	25.30 ± 0.90^b^	19.80 ± 0.11^a^	541.50 ± 3.00^c^
D100%	31.70 ± 0.91^a^	53.70 ± 0.91^c^	23.70 ± 2.70^b^	14.70 ± 0.91^c^	564.60 ± 5.10^b^

The mean is followed by the standard deviation (SD). Values in the same column with the same superscripts do not differ significantly (P˃0.05), (n = 5).

### 3.5 Amino acid content of the investigational diets and fish

Table 2 summarizes the amino acid profile of dietary treatments. Lysine content ranged from 2.11% to 3.21% of the total amino acids in numerous diets. The percentage of lysine decreased with increasing levels of PBM substitution. The diet with 100% PBM (D100%) had the lowest lysine content (2.11%), while the control diet (D0%) had the highest (3.21%). Only the D100% diet contained lysine levels below the requirement for European sea bass (Table 2). However, the essential amino acid (EAA) composition of the fish did not differ significantly, particularly in terms of the whole-body contents of methionine, arginine, threonine and histidine, when comparing fish fed the control diet (D0) to those fed replacement diets up to 50% PBM. Lysine, arginine, leucine, and valine were the predominant EAAs in fish fed PBM diets, while glutamic and glycine were the most abundant non- EAAs in these fish (Table 6).

**Table 6:** Amino acid profile (g kg^−1^ protein of dry weight) of European sea bass (*D. labrax L*) fed with PBM as a substitution of fishmeal for 12 weeks.

	Level of replacement (%)				
Amino acids	D0%	D25%	D50%	D75%	D100%
**Essential Amino Acids**					
Arginine	65.04 ± 0.26^a^	64.77 ± 0.68^a^	64.45 ± 0.28^a^	63.49 ± 0.19^a^	58.38 ± 0.12^b^
Histidine	51.57 ± 0.08^a^	51.25 ± 0.15^ab^	50.64 ± 0.10^ab^	50.48 ± 0.26^b^	48.43 ± 0.40^c^
Isoleucine	46.26 ± 0.25^a^	43.49 ± 0.24^b^	43.12 ± 0.45^b^	43.26 ± 0.34^b^	36.32 ± 0.20^c^
Leucine	64.32 ± 0.39^a^	62.40 ± 0.33^b^	60.58 ± 0.60^c^	60.29 ± 0.14^c^	48.34 ± 0.20^d^
Lysine	74.96 ± 0.51^a^	72.83 ± 0.36^b^	72.52 ± 0.41^b^	72.02 ± 0.24^b^	62.36 ± 0.16^c^
Methionine	32.38 ± 0.08^a^	31.73 ± 0.16^ab^	31.68 ± 0.13^ab^	31.29 ± 0.10^b^	25.54 ± 0.28^c^
Phenylalanine	35.37 ± 0.27^a^	33.64 ± 0.30^b^	32.60 ± 0.18^bc^	32.44 ± 0.10^c^	27.49 ± 0.27^d^
Threonine	41.51 ± 0.28^a^	41.17 ± 0.05^ab^	40.45 ± 0.12^ab^	40.09 ± 0.18^b^	38.32 ± 0.47^c^
Valine	54.19 ± 0.13^a^	52.24 ± 0.17^b^	49.73 ± 0.07^c^	41.93 ± 0.06^d^	37.51 ± 0.23^e^
**Non-Essential Amino Acids**					
Aspartic acid	74.91 ± 0.24^a^	72.80 ± 0.46^b^	72.02 ± 0.24^bc^	70.93 ± 0.33^c^	56.11 ± 0.36^d^
Serine	32.62 ± 0.14^a^	31.52 ± 0.17^b^	30.78 ± 0.17^b^	29.58 ± 0.21^c^	25.72 ± 0.09^d^
Glycine	87.13 ± 0.31^a^	83.42 ± 0.04^b^	81.66 ± 0.10^c^	81.09 ± 0.09^c^	71.42 ± 0.21^d^
Alanine	74.31 ± 0.15^a^	71.13 ± 0.55^b^	69.42 ± 0.62^bc^	67.54 ± 0.55^c^	63.38 ± 0.16^d^
Cystine	8.58 ± 0.14^a^	6.22 ± 0.34^b^	5.08 ± 0.48^b^	4.87 ± 0.35^b^	2.14 ± 0.09^c^
Glutamic acid	94.57 ± 0.14^a^	82.64 ± 0.10^b^	81.21 ± 0.44^b^	80.47 ± 0.28^b^	74.44 ± 0.33^c^
Proline	33.91 ± 0.35^a^	30.76 ± 0.39^b^	29.90 ± 0.60^bc^	28.83 ± 0.32^c^	26.19 ± 0.29^d^
Tyrosine	19.25 ± 0.39^a^	17.29 ± 0.18^b^	16.25 ± 0.31^b^	14.80 ± 0.37^c^	11.32 ± 0.26^d^

The mean is followed by the standard deviation (SD). Values in the same column with the same superscripts do not differ significantly (P˃0.05), (n = 5).

### 3.6 Histological analysis

**Fig. 3:**
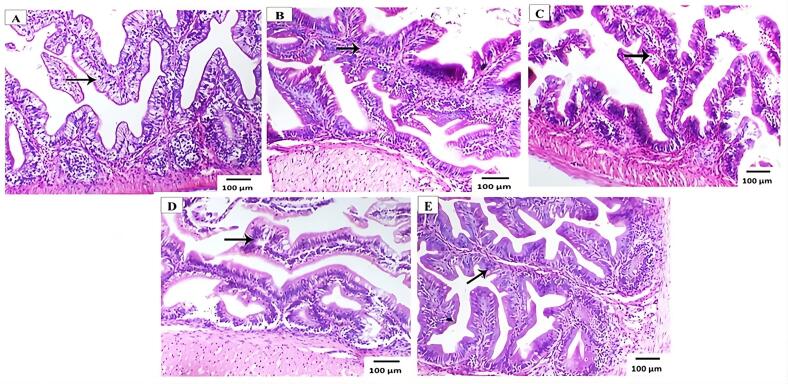
Light photomicrograph of intestine sections of European sea bass (*D. labrax L*) fed on control diet 0% (A), diet 25% (B), diet 50% (C), diet 75% (D), diet (E) 100% replacement with PBM, showing normal villi structure in all treatments (H&E, X100), *n* = 25.

**Fig. 4:**
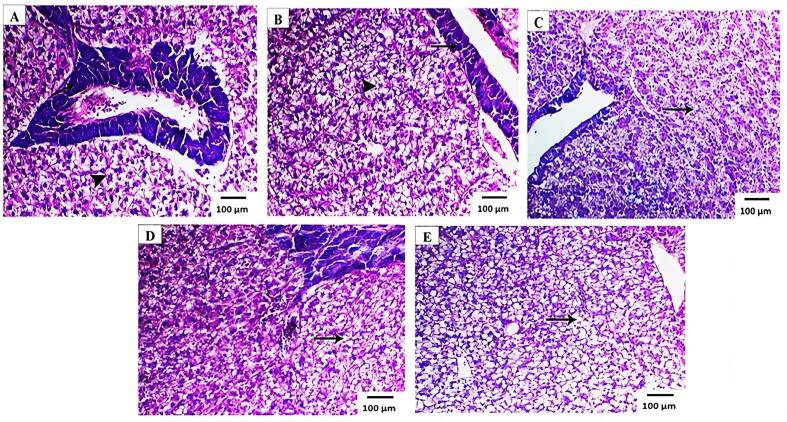
Light photomicrograph of liver sections of European sea bass (*D. labrax L*) fed on control diet 0% (A), diet 25% (B), diet 50% (C), diet 75% (D), diet (E) 100% substitution with PBM showing mild vacuolation of hepatocytes in diet 50% while diet 75 &100% PBM showing pre-pancreatic fatty degeneration of hepatocytes. Also, (A) arrows showing that fish fed control diet (0% PBM) possesses more hepatocyte cytoplasmic vacuolation and (B, C, D, E) showing that fish fed (25%, 50%,75% and 100% PBM) possesses less hepatocyte cytoplasmic vacuolation, n = 25.

On the termination of the trial, the microscopic structure of the European sea bass intestine and liver was investigated for histological analysis, with results presented in Figs. (3 & 4) and Table 7. The study found that the length, width and absorption area of intestinal microvilli significantly (*p*˃0.05) improved with increasing levels of fishmeal replacement, reaching their maximum at the 50% PBM diet. This enhancement in microvilli morphology suggests a reduced food passage time (Table 7). Furthermore, goblet cells and apical epithelial vacuoles were consistently observed across all diet groups, although enterocyte morphology varied in fish fed different PBM concentrations. Notably, the submucosal layer of fish fed higher PBM diets contained a significantly greater number of eosinophil cells as shown in Fig. 3. Light photomicrographs of European sea bass liver sections revealed that fish fed the control diet (0% PBM) exhibited minimal hepatocyte cytoplasmic vacuolation, implying a lower glycogen accumulation. In contrast fish fed diets with varying PBM concentrations (25%, 50%, 75%, and 100%), displayed increased hepatocyte cytoplasmic vacuolation, indicating higher glycogen and lipid vacuolization (Fig. 4).

**Table 7:** Morphometric analysis of intestinal tract of European sea bass (*D. labrax L*) fed with PBM as a replacement of fishmeal for 12 weeks.

Items	D0%	D25%	D50%	D75%	D100%
Villi length (μm^2^)	263.22 ± 5.82 ^ab^	272.56 ± 6.42 ^ab^	316.22 ± 2.73 ^a^	301.00 ± 6.71 ^ab^	155.56 ± 10.51 ^b^
Villi width (μm^2^)	36.00 ± 1.62 ^ab^	35.00 ± 2.10 ^b^	51.00 ± 1.40 ^a^	48.22 ± 2.22 ^ab^	21.22 ± 2.41 ^c^
Area of absorption (μm^2^)	9475.92 ± 472.6^ab^	9539.60 ± 1313 ^ab^	16,127.22 ± 1116.2^a^	14,514.22 ± 1278.6^a^	3300.98 ± 701.2^b^

The mean is followed by the standard deviation (SD). Values in the same column with the same superscripts do not differ significantly (P˃0.05), n = 5 × 5 = 25.

## 4 Discussion

### 4.1 Growth efficiency

Based on our analysis of growth parameters, we recommend that the maximum substitution level of poultry by-product meal (PBM) for fishmeal (FM) should be up to 50% for juvenile European sea bass. This recommendation aligns with previous studies on various marine finfish species, which have reported effective substitution levels of 50% PBM. In the current study, we observed a decrease in weight gain in European sea bass when PBM was substituted for FM at a rate of 60% (Srour, Essa, Abdel-Rahim, & Mansour, 2016). European sea bass exhibit a higher capacity to digest PBM compared to other marine fish, which allows them to thrive on diets with high levels of PBM substitution for FM. This enhanced ability might be attributed to the presence of different digestive enzymes such as amylase, protease, and lipase which facilitate the absorption of PBM inEuropean sea bass. Additionally, more research is needed to evaluate the long term impacts of PBM on the growth performance and morphological characteristics of candidate species. For instance, in red drum (*Sciaenops ocellatus*), no statistically significant differences in growth performance were observed when 66.7% FM was replaced by PBM (Wang et al., 2006). In contrast, black sea turbot, *Psetta maeotica* exhibited decreased final weight and specific growth rate when diets replaced more than half of the FM protein with PBM (Yang et al., 2004). It is crucial to note that substituting FM with alternate ingredients like PBM should not negatively impact fish welfare or growth, which has been increasingly concerning over the decades (Saraiva, Costa, Serrão, Cruz, & Eiras, 2015). Balanced dietary essential amino acids (EAA) in PBM-based diets are key determinants in improving growth efficiency in many aquatic animals (Galkanda-Arachchige, Wilson, & Davis, 2020). Similarly deficiencies in methionine, phenylalanine, isoleucine, lysine, and histidine have been shown to reduce the production efficiency in sea bream (*Acanthoparus schlegelii*) juveniles when supplemented with high levels of PBM (Irm et al., 2020). Given that this study evaluated at four different levels of PBM, higher levels of FM replacement may be possible in juvenile European sea bass diets. Further research is warranted to optimize PBM inclusion rates while ensuring optimal growth and health of the fish.

### 4.2 Survival

The survival rate in this trial remained high throughout ranging from 80 to 95% with significant changes across the treatments, while the significantly (*p* ≤ 0.5) lowest value was noted at 100% PBM level. The appropriate partial replacement of PBM appears to be up to 75% in terms of growing performance and feed use in European sea bass (*D. labrax* L) with no negative effects on survival rate. The poor survival rate at 100% PBM level may also be likened to amino acids imbalance. Diverse researchers, including Hansen, Karlsen, Rosenlund, Rimbach, and Hemre (2007) for Atlantic cod, Bureau, Harris, and Cho (1999) for rainbow trout, and Kureshy et al. (2000) for red drum obtained comparable observations. Similarly, 100% PBM diets had superior MUFA content and inferior quantities of PUFA, especially n-3 LC-PUFA and EPA content, which may be held accountable for the detrimental effects on survival, growth, and feed consumption. Additionally, polyunsaturated fatty acids (PUFAs) were described as necessary for get the best growth and survival of aquatic animals (Galkanda-Arachchige et al., 2020). There are various impacts of inclusion PBM levels on the growth and biometry indications, maybe owing to use of differing fish species, aquatic systems, palatability and digestibility of PBM (Chaklader et al., 2020).

### 4.3 Feed utilization efficiency

Herein, significant changes in apparent feed use were seen across the groups demonstrating that palatability could be an issue for juvenile European sea bass. Contrary to our statement, Yigit et al. (2006) reported that replacing up to 50% of PBM in diets with black sea turbot (*P. maeotica*) had insignificant impact on PER. We also noted that the reduction in feed utilization at higher level of PBM (D100%) might be attributed to low methionine content present in PBM than FM. Previously, several studies have observed poor growth and feed utilization in different fish species due to methionine deficiency (El-Ouny et al., 2023). Moreover, when black sea turbot, *P. maeotica* was fed diets with 75 or 100% FM substituted by PBM, Yigit et al. (2006) observed a decrease in feed consumption, but no significant change was observed at 50% PBM.

### 4.4 Whole body composition

The dry matter composition of the whole fish body differed significantly across the treatments in this study. Notably, the whole-body lipid content in fish fed a 75% PBM diet was insignificantly different from that of fish fed the FM diet. This finding is consistent with previous studies on gilthead sea bream (*Sparus aurata*) and rainbow trout (*Oncorhynchus mykiss*), where substituting FM with PBM did not result in substantial changes in whole-body lipid content (Carter et al., 2012; Chaklader, Siddik, Fotedar, & Howieson, 2019). In this research, the highest protein content was observed in fish fed a 50% PBM diet, while those fed a 100% PBM diet had lower protein content compared to other groups. This variation could be attributable to the different developmental phases of the fish studied. Contrary to our results, Srour et al. (2016) reported no significant difference in carcass crude protein in European sea bass when up to 56% FM was replaced by plant-based meal. Similar findings have been reported by several authors for different fish species fed PBM diets replacing FM. For instance, Jo et al. (2017) found no significant differences in carcass crude protein in rainbow trout, and Damusaru et al. (2019) reported comparable results for Japanese eel (*Anguilla japonica*).

### 4.5 Amino acid composition of the diet

Amino acids play a crucial role in the metabolism of protein and overall health of aquatic animals (El-Ouny et al., 2023). In the present study, lysine percentage was found to be lower in the D100% PBM diet compared to the FM (D0) and PBM diets with lower inclusion levels (Table 2). The demands of lysine for the grow-out phase in European sea bass are well-documented by Gaber, Zaki, Nour, and Salem (2015). When lysine is deficient in the diet, it adversely affects fish growth because lysine is an essential limiting amino acid for many fish species (Hardy & Barrows, 2002). Ai et al. (2006) reported that the poor growth performance in cuneate drum and turbot fed a D100% PBM diet might be due to a lack of lysine content. Similarly, García-Pérez, Cruz-Valdez, Ramírez-Martínez, Villarreal-Cavazos, and Gamboa-Delgado (2018) found that low lysine levels in the diet of gilthead sea bream (*Sparus aurata*) with 75% PBM or more substitution partly caused decreased growth.

Kureshy et al. (2000) and Yigit et al. (2006) also linked reduced growth in fish fed PBM diets to low lysine and n-3 fatty acids (HUFA) content and variability in PBM quality. In this study, dietary histidine concentration ranged from 0.90 to 1.00% of the total amino acids, with histidine content diminishing as PBM substitution levels increased. The PBM (1.50%) and FM diets had similar histidine content (1.60%). However, all diets had less histidine content than the fish required (1.60% of the total amino acids). Moreover, it was found that European sea bass fed PBM diets did not show significant changes in overall performance. Lysine was identified as a key essential amino acid (EAA) among the total amino acids, while glutamic acid and glycine were the predominant non-essential amino acids. Damusaru et al. (2019) reported comparable results for *Anguilla japonica*, where lysine was also found to be the predominant EAA. This is likely due to the high protein content of PBM, making it a more available and economical ingredient compared to fishmeal (Gupta et al., 2020).

### 4.6 Histological structure of fish

Assessing the histological structure of fish liver is critical for studying the impacts of various raw ingredients used in aquafeed (Eissa et al., 2022). In the present study, histological sections of liver revealed that the fish fed control diet (0% PBM) had less hepatocyte cytoplasmic vacuolation, which could be attributed to the presence of less glycogen. Conversely, the liver sections of fish fed diets containing varied concentrations of PBM diets exhibited high hepatocyte vacuolation. Zhou et al. (2020) found rising steatosis in hybrid grouper hepatocytes when fish were fed 50–70% PBM diets as a substitution level for fishmeal. However, the hepatocytes of *Salmo salar* (Hartviksen et al., 2014) and *Oreochromis niloticus* (Aydin, Gumus, & Balcl, 2015) did not exhibit any enhanced vacuolization or modifications as a result of the complete substitution of FM by PBM.

Comparatively, Glencross, Evans, Hawkins, and Jones (2004) found small lipid droplets in the liver of rainbow trout, which they believe might be related to the diet's deficient energy retention due to high amounts of yellow lupine kernel meal. Plant protein substitution has no effect on the liver function and morphology of sharp snout sea bream, *Diplodus puntazzo* (Mérida, Tomás-Vidal, Martínez-Llorens, & Cerdá, 2010). Similar findings were made by Hu et al. (2013), who found that high-level substitution up to 60–80% PBM caused hepatocytes to enlarge and produced apparent hepatic steatosis. Moreover, in the current study, liver sections of European sea bass also showed pre-pancreatic fatty degeneration of hepatocytes in fish containing 75 and 100% PBM diet. It is possible that liver fat abundance is the consequence of an excess of PMB diets that exceeds the liver's physiological ability to metabolize them, resulting in steatosis (Psofakis et al., 2021).

The intestinal tract is an innate immunity organ in fish that aids in the digestive process, feed utilization, and nutrient absorption while also acting as a protective mechanism against microbial species (Eissa et al., 2023). Assessing intestinal morphology in response to food changes is critical for evaluating fish welfare and health (Abdel-Tawwab et al., 2023). Nutritional absorption and assimilation are related to immune function and intestinal morphological characteristics, specifically microvillus diameter and structure (Kord et al., 2022). In this study, the inclusion of PBM caused few intestinal histological alterations in seabass. All fish groups except D100% PBM confirmed apparent enterocytes with plentiful eosinophil cells, goblet cells, and apical epithelial vacuoles exhibited along the whole intestine of fish. Goblet cells contribute to the health and nutrition of fish by secreting mucus that protects the epithelium and lubricates undigested items as they move into the rectum (Berillis & Mente, 2017). Additionally, apical epithelial vacuoles are essential intestinal structural elements responsible for nutrient engagement (Khalil et al., 2023). Similarly, the intestinal histology of Atlantic salmon fed on high amounts of PBM in place of dietary FM was reported to be unaffected (Hu et al., 2016).

## 5 Conclusion and recommendations

The present study demonstrates that poultry by-product meal (PBM) can replace up to 50% of fishmeal in the diet of European sea bass (*Dicentrarchus labrax*) without negatively affecting growth rate, body composition, or liver and intestine morphology. Given the significantly lower cost of PBM compared to fishmeal, this substitution presents a cost-effective alternative. However, further research, including long-term feeding trials, is required to confirm these findings. Therefore, the adoption of PBM-based diets in place of fishmeal is strongly recommended for the sustainable production of marine fish.

## CRediT authorship contribution statement

**Yasser Marzouk:** Writing – original draft, Methodology, Investigation, Formal analysis, Data curation. **Magdy M. Gaber:** Writing – original draft, Validation, Resources, Project administration, Conceptualization. **Ishtiyaq Ahmad:** Writing – review & editing, Writing – original draft, Visualization, Formal analysis, Conceptualization. **Imtiaz Ahmed:** Writing – review & editing, Visualization, Validation, Investigation. **Mohammed F. El Basuini:** Writing – review & editing, Visualization, Resources, Methodology, Formal analysis, Data curation. **Mohamed Abdullah Zaki:** Writing – review & editing, Resources, Methodology, Formal analysis, Data curation. **Abd-Elaziz M. Nour:** Writing – review & editing, Visualization, Resources, Methodology, Formal analysis, Data curation. **Eman M.H. Labib:** Writing – review & editing, Resources, Methodology, Data curation. **Hala Saber Khalil:** Writing – review & editing, Writing – original draft, Validation, Resources, Methodology, Formal analysis, Data curation, Conceptualization.

## Declaration of competing interest

The authors declare that they have no known competing financial interests or personal relationships that could have appeared to influence the work reported in this paper.

## Data Availability

The raw data were produced are available upon request from the corresponding author.
